# P139: Clean hands fact-finding mission 2012 to Afghanistan: good intentions, minor results, why?

**DOI:** 10.1186/2047-2994-2-S1-P139

**Published:** 2013-06-20

**Authors:** D Pittet, K-W Stahl

**Affiliations:** 1 Infection Control Programme, University of Geneva Hospitals, Geneva, Switzerland; 2 Waisenmedizin e V, Freiburg, Germany

## Introduction

The Balkh Province Civil Hospital (BBCH), Mazar-e-Sharif, Afghanistan, has undergone a €20 million major reconstruction financed by KfW, Germany, a government-owned development bank, and is used as a teaching hospital by the Balkh Medical Faculty. In 2009, the German Government sponsored the modernisation of the BBCH leishmania centre (cutaneous leishmania is endemic in Afghanistan) with €0.1 million, which allowed to run wound-healing trials. In this outpatient clinic, wound-healing impairment typical of cutaneous leishmania may be aggravated if combined with neglected basic hand hygiene, particularly as a moist environment must be maintained for healing.

## Methods

We conducted a fact-finding mission in April 2012 to Afghanistan to evaluate hand hygiene practices in selected hospitals, including the BBCH. Inspection tours to 5 Afghan hospitals were organized by the WHO Kabul Office. Lectures were given to medical students and hospital physicians to raise awareness and understanding of the importance of hand hygiene.

## Results

On 11 April 2012, Afghanistan became the 130^th^ nation of the 194 UN Member States to sign the pledge of the WHO Global Patient Safety Challenge “Clean Care is Safer Care”. WHO posters promoting the “My 5 moments for hand hygiene” concept were displayed in most hospitals, but alcohol-based handrub was only available in 2 hospitals (Rabi Balkhi, Kabul and ANA Hospital, Mazar). This prompted the idea of installing a local plant for ethanol production from wheat at the Agricultural Faculty of the Balkh University to be financed by the German Ministry of Cooperation (BMZ) as a female gender project with refilling of handrub bottles according to the WHO Mali pilot site model. A requirement of BMZ funding was a written authorisation from the provincial governor for bio-ethanol production for medicinal purposes and his assurance to take responsibility for safe storage, but after more than 1 year this has not been forthcoming.

## Conclusion

In a failed state environment, developmental aid in health care is not only facing ignorance and eventual religious constraints, but also corruption and a problem of smuggling, which impedes an easy solution to implement hand hygiene.

## Disclosure of interest

None declared

